# The lipid phosphatase Synaptojanin 1 undergoes a significant alteration in expression and solubility and is associated with brain lesions in Alzheimer’s disease

**DOI:** 10.1186/s40478-020-00954-1

**Published:** 2020-06-03

**Authors:** Kunie Ando, Marième Ndjim, Sabrina Turbant, Gaëlle Fontaine, Gustavo Pregoni, Luce Dauphinot, Zehra Yilmaz, Valérie Suain, Salwa Mansour, Michèle Authelet, Robert De Dekker, Karelle Leroy, Benoît Delatour, Brain Bank NeuroCEB Neuropathology Network, Franck Letournel, Marie-Laure Martin-Négrier, Françoise Chapon, Catherine Godfraind, Claude-Alain Maurage, Vincent Deramecourt, David Meyronnet, Nathalie Streichenberger, André Maues de Paula, Valérie Rigau, Fanny Vandenbos-Burel, Charles Duyckaerts, Danielle Seilhean, Susana Boluda, Isabelle Plu, Serge Milin, Dan Christian Chiforeanu, Annie Laquerrière, Béatrice Lannes, Charles Duyckaerts, Marie-Claude Potier, Jean-Pierre Brion

**Affiliations:** 1 Laboratory of Histology, Neuroanatomy and Neuropathology, ULB Neuroscience Institute, Université Libre de Bruxelles (ULB), 808, route de Lennik (Bldg G), 1070 Brussels, Belgium; 2 Laboratoire de Neuropathologie Escourolle, Hôpital de la Pitié-Salpêtrière, AP-HP, Paris, France; 3 ICM Institut du Cerveau, CNRS UMR7225, INSERM U1127, UPMC, Hôpital de la Pitié-Salpêtrière, Paris, France

**Keywords:** Alzheimer’s disease, SYNJ1, Amyloid β, Tau, Neurofibrillary tangles, Hirano bodies, Phosphatidylinositol

## Abstract

Synaptojanin 1 (SYNJ1) is a brain-enriched lipid phosphatase critically involved in autophagosomal/endosomal trafficking, synaptic vesicle recycling and metabolism of phosphoinositides. Previous studies suggest that *SYNJ1* polymorphisms have significant impact on the age of onset of Alzheimer’s disease (AD) and that SYNJ1 is involved in amyloid-induced toxicity. Yet SYNJ1 protein level and cellular localization in *post-mortem* human AD brain tissues have remained elusive. This study aimed to examine whether SYNJ1 localization and expression are altered in *post-mortem* AD brains. We found that SYNJ1 is accumulated in Hirano bodies, plaque-associated dystrophic neurites and some neurofibrillary tangles (NFTs). SYNJ1 immunoreactivity was higher in neurons and in the senile plaques in AD patients carrying one or two *ApolipoproteinE (APOE) ε4* allele(s). In two large cohorts of *APOE*-genotyped controls and AD patients, *SYNJ1* transcripts were significantly increased in AD temporal isocortex compared to control. There was a significant increase in *SYNJ1* transcript in *APOEε4* carriers compared to non-carriers in AD cohort. SYNJ1 was systematically co-enriched with PHF-tau in the sarkosyl-insoluble fraction of AD brain. In the RIPA-insoluble fraction containing protein aggregates, SYNJ1 proteins were significantly increased and observed as a smear containing full-length and cleaved fragments in AD brains. In vitro cleavage assay showed that SYNJ1 is a substrate of calpain, which is highly activated in AD brains. Our study provides evidence of alterations in *SYNJ1* mRNA level and SYNJ1 protein degradation, solubility and localization in AD brains.

## Introduction

Alzheimer disease (AD) is neuropathologically characterized by extracellular amyloid plaques composed of amyloid β (Aβ) peptides and intracellular neurofibrillary tangles (NFTs) constituted of microtubule associated protein tau. AD lesions are tightly related to severe misregulation of cytoskeletal proteins such as microtubule or actin. Hirano bodies, eosinophilic crystal-like structures, are frequently observed in pyramidal neurons of the CA1 area of the Ammon’s horn of AD brains [27]. Hirano bodies are composed of intracellular aggregates of actin, actin-binding proteins, and tau [24]. Smaller inclusions positive for actin-depolymerizing factor ADF/cofilin often occur in linear arrays in AD brains and transgenic AD models [41].

Several studies have shown that lipid phosphatase Synaptojanin1 (SYNJ1) is profoundly involved in human neurodegenerative diseases such as AD, early onset Parkinson’s disease (PD) and Down syndrome (DS). SYNJ1, originally described as an inositol-5 phosphatase enriched in axon terminals, dephosphorylates phosphatidylinositol 4,5-bisphosphate (PIP_2_) and phosphatidylinositol 3,4,5-trisphosphate (PIP_3_) at position 5 [39, 60]. SYNJ1 is an essential protein involved in autophagosomal and endosomal trafficking [25, 57] and participates in synaptic vesicle recycling [20]. Single nucleotide polymorphisms of *SYNJ1* have significant impact on the age of onset of AD [42]. Human *SYNJ1* mutations have been reported in familial PD: R268G substitution of *SYNJ1* SAC1 domain was identified in early onset familial PD [29, 48, 50]. Homozygous R268G substitution causes Parkinsonian phenotype in knock-in mice [16] and causes presynaptic autophagy defects in flies [57]. *SYNJ1* maps to chromosome 21 and SYNJ1 expression is increased in the cortex and in lymphoblastoid cell lines and fibroblasts of individuals with DS [1, 7, 18, 19]. SYNJ1 expression is exacerbated in old individuals with Down syndrome with AD-like neuropathological lesions (DSAD) [38]. Whereas excessive Synj1 expression leads to memory deficits in rodent [59], homozygous *Synj1* knockout mice are lethal [20] and a rare human homozygous nonsense mutation in *SYNJ1* caused epilepsy and severe tau pathology in a young child [22].

Despite significant implication of SYNJ1 in AD, its localization and expression levels remain unclear in AD brains. There are several controversies as to whether SYNJ1 expression is increased or decreased in AD brains. One study has shown that SYNJ1 protein level is decreased in AD [38] while other studies have reported a significant increase of SYNJ1 in AD brains [42], in association with the *APOEε4* allele [61]. In this study, we aimed to analyse the localization and expression level of SYNJ1 protein in human *post-mortem* brain tissues of non-demented control and AD cases. We found that SYNJ1 immunoreactivity was associated with dystrophic neurites surrounding amyloid plaques where SYNJ1 and the presynaptic marker Synaptophysin were partially colocalized. SYNJ1 immunoreactivity was also detected in actin positive Hirano bodies and in a proportion of the NFTs. *SYNJ1* transcripts were upregulated in AD brains, with higher levels in AD patients bearing *APOEε4* allele(s) compared to those bearing no *APOEε4* allele. SYNJ1 protein was predominantly detected in highly insoluble fractions of AD brains. This study demonstrates that SYNJ1 is significantly mislocalized and misregulated in AD brains.

## Materials and methods

### Antibodies

Five anti-Synaptojanin1 antibodies were used in this study (Supplementary Table 1, online resource). Rabbit polyclonal anti-SYNJ1 (HPA011916) was purchased from Sigma. Mouse monoclonal anti-SYNJ1 (BD612249, sc-32,770, TA309245) antibodies were purchased from BD transduction, Santa Cruz Biotechnology and Origene, respectively. Rabbit polyclonal anti-SYNJ1 ab19904 antibody was purchased from Abcam. Mouse monoclonal anti-Flag M2 (F3165), and mouse monoclonal anti-actin antibodies (A5441) were purchased from Sigma. Mouse monoclonal anti-tau antibody recognizing pSer396/Ser404 tau (PHF1) was kindly provided by Dr. Peter Davies (Albert Einstein College of Medicine, NY). Mouse monoclonal anti-Synaptophysin (SY38) was purchased from abcam.

### Human brain tissues

Samples from the temporal superior T1 isocortex and hippocampus were obtained from AD and age-matched non-demented control subjects. AD cases were diagnosed according to the National Institute of Aging and Reagan Institute Criteria [9] and scored by neuropathological staging for tau and amyloid pathologies [12, 56]. AD cases including two FAD cases with *Amyloid Precursor Protein (APP)* or *Presenilin1* (*PSEN1*) mutations and one DSAD were all scored as Braak’s stage V or VI (Supplementary Table 2, online resource). Control cases were non-demented individuals who died without known neurological disorders. The mean ages and *post-mortem* delays of control cases and of AD patients were not significantly different. Average age at death was 76.8 +/− 1.5 and 75.4 +/− 1.5 years for control (*n* = 43) and AD (*n* = 51) cases respectively (mean +/− SEM) (*p* = 0.54). Average *post-mortem* delays were 21.8 +/− 2.8 h and 20.1 +/− 1.8 h for control and AD cases (mean +/− SEM) (*p* = 0.59). *APOE* genotype was determined for the cases with an informed consent for genetic study using PCR amplification for genomic DNA and sequencing as described [55].

Non-demented control and AD individuals were enrolled in a brain donation program of the national network of Brain Bank, GIE NeuroCEB, organized by a consortium of Patients Associations. An explicit consent had been signed by the patient or by the next of kin, in the name of the patient. The project was approved by the scientific committee of the Brain Bank. The whole procedure of the Brain Bank has been reviewed and accepted by the Ethical Committee “Comité de Protection des Personnes Paris Ile de France VI” and has been declared to the Ministry of Research and Higher Education as requested by the French law. Some cases were obtained from ULB LHNN brain bank (BB190052) and were studied in compliance and following approval of the Ethical Committee of the Medical School of the Free University of Brussels.

### Animals

The 5XFAD double transgenic mice co-express the human amyloid precursor protein (APP695) carrying the Swedish, Florida, and London mutations and the human PSEN1 carrying the M146L and L286V mutations under thy-1 promoter (Tg6799 line) [47]. The 5XFAD mice were maintained on C57Bl/6 J genetic background and only heterozygous transgenic mice were used for this study. Paraffin embedded brain sections of 12 month-old 5XFAD mice (*n* = 5) were analysed in this study [33]. Wild-type Wistar rat embryos at embryonic day 17 (E17) were prepared as previously described [34]. All studies on animals were performed in compliance and following the approval of the ethical committee for the care and use of laboratory animals of the Medical School of the Free University of Brussels.

### Immunohistochemistry

After formaldehyde fixation (10% buffered formalin), brain tissues were paraffin embedded and sliced in 7 μm thick sections. DAB staining was performed as previously described [2]. Double immunofluorescence labelling was performed using Tyramide-FITC kit (NEL701A, Perkin Elmer), using a goat anti-rabbit antibody conjugated with biotin (Vector Laboratories, BA-1000) for SYNJ1 detection. Mouse monoclonal antibodies were detected using a goat anti-mouse antibody conjugated to Alexa 568 (A-11031, Invitrogen). Slides were mounted with Fluoromount-G (Southern Biotech) and immunofluorescence labelling was observed with an upright confocal microscope (Olympus Fluoview Fv1000) or with an Axiovert 200 M microscope (Zeiss) equipped with an ApoTome system (Zeiss). For quantitative analysis, SYNJ1 positive neurons and dystrophic neurites in hippocampal CA1–2 pyramidal layer were analysed at 40X images by thresholding analyses using NIH ImageJ as previously reported [58].

### Cell cultures and immunocytochemistry

HEK 293 cells were grown in DMEM medium F12 supplemented with 10% foetal bovine serum, 100 IU penicillin and 100 μg of streptomycin. For immunocytochemistry, HEK 293 cells grown on PLL-coated cover glasses were transfected with Flag-SYNJ1 1–145 human neuronal isoform (145 kDa) DNA plasmid, a generous gift from Prof. Pietro De Camilli, using Lipofectamine 2000 (Life technologies). Twenty-four hours after transfection, cells were rinsed and fixed (4% paraformaldehyde and 4% sucrose in PBS) for 20 min at room temperature followed by rinses and quenching with 10 mM NH_4_Cl in PBS for 10 min. The cells were permeabilized with 0.3% BSA and 0.05% Saponin in PBS at 37 °C for 45 min. After overnight incubation with primary antibodies, the cells were rinsed and incubated with goat anti-rabbit IgG conjugated with Alexa 488 and goat anti-mouse IgG conjugated with Alexa 568 (A-11034 and A-11031, Life technologies).

### RNA extraction and quantitative PCR (qPCR) for *SYNJ1* mRNA

Total RNAs from human T1 isocortex were extracted using Nucleospin RNA II kit (Macherey Nagel, Duren, Germany). The quality and quantity of each RNA preparation were assessed on an Agilent 2100 Bioanalyzer with RNA 6000 NanoChips (Agilent Technologies, Santa Clara, CA, USA). Briefly, RNAs (500 ng) were individually reverse-transcribed into cDNAs for 10 min at 25 °C, then 2 h at 42 °C followed by 5 min at 85 °C using the SensiFAST cDNA synthesis kit (Bioline-Meridian Bioscience, London, UK) according to the manufacturer’s instructions. qPCR gene expression assays were performed in a LC96 system (Roche), in the presence of 1X Lightcycler® 480 Probes Master mix (Roche, France), 200 nM of each primer and 100 nM of specific hydrolysis probe (designed with Universal Probe Library, Roche Applied Science): SYNJ1 5′ gtgttctgcggttaaatcttg 3′ (forward), 5’tcttgaatttttccaacagacatac 3′ (reverse), PolR2A 5′ caagttcaaccaagccattg 3′ (forward), 5′ gtggcaggttctccaagg 3′ (reverse) pPib 5′ ttcttcataaccacagtcaagacc 3′ (forward), 5′ accttccgtaccacatccat 3′ (reverse) and RNF4 5’ctcaggtactgtcagttgtc 3′(forward) and 5’cgatgagacgtccattctg 3′ (reverse).

SYNJ1 expression was normalized to Peptidylprolyl Isomerase B (pPib), Ring Finger Protein 4 (RNF4) and DNA-directed RNA polymerase II subunit RPB1 (PolR2A).

### Preparation of brain homogenates for biochemical analysis

About 200 mg of frozen T1 isocortex was homogenised as reported [2, 4] in 10 volumes of ice-cold RIPA buffer containing 50 mM Tris pH 7.4 containing 150 mM NaCl, 1% NP-40, 0.25% sodium deoxycholate, 5 mM EDTA, 1 mM EGTA, Roche complete protease inhibitors, 1 mM PMSF, and phosphatase inhibitor cocktail 2, (Sigma, P-5726) and incubated for 60 min at 4 °C on a rotator. One hundred microliter of the total homogenate was supplemented with Laemmli buffer, sonicated on ice and analysed as the total fraction. The rest of the total homogenates was centrifuged (20,000 x g for 20 min at 4 °C) and the supernatant was used as a RIPA soluble fraction. The RIPA-insoluble pellet was re-suspended by sonication in 5-fold volume of 8 M urea containing protease and phosphatase inhibitors and incubated for 30 min at room temperature on a rotator. The mixture was centrifuged at 20,000 x g at 4 °C for 30 min. The supernatant was used as RIPA insoluble fraction. For each fraction, protein concentrations were estimated by the Bradford method (Bio-Rad) before adding Laemmli buffer.

Sarkosyl fractionation was carried out as previously described [4, 14, 26]. To 1 ml of RIPA soluble fraction of brain lysates at a protein concentration of 2 mg/ml, 10 mg of N-lauroylsarcosine sodium salt (Sigma, L-5125) was added to reach a final concentration of 1% (w/v). The lysates were then incubated at room temperature for 30 min with a mild agitation followed by an ultracentrifugation at 180,000 x g for 30 min at 4 °C. The sarkosyl-soluble supernatant was removed, and sarkosyl-insoluble pellets were rinsed briefly with 500 μl of 50 mM Tris-HCl (pH 7.4) and re-suspended in 200 μl of 50 mM Tris-HCl (pH 7.4) by vigorous pipetting. Sarkosyl-insoluble fractions were analysed by western blotting (WB) and transmission electron microscopy as previously described [3, 4].

### WB

Tissue samples (20 μg/lane) were run in 7.5% Tris-Glycine gels and transferred onto nitrocellulose membranes (sc-3724, Santa Cruz Biotechnology). The nitrocellulose membranes were blocked in 10% (w/v) semi fat dry milk in TBS (Tris 0.01 M, NaCl 0.15 M, pH 7.4) for 1 h at room temperature and were incubated with primary antibodies overnight followed by rinses and an incubation with anti-rabbit (#7074, Cell Signalling Technology, Bioké) or anti-mouse (A6782, Sigma) immunoglobulin conjugated to horseradish peroxidase. After several rinses, the membranes were incubated with SuperSignal West Pico Substrate (Pierce) and were exposed to an X-ray film (Pierce) or to a DARQ-7 CCD cooled camera (Vilber-Lourmat) in a SOLO 4S WL system. Levels of optical density (OD) of protein signals were estimated by densitometry analysis using the NIH ImageJ program. Anti-β-actin immunoblots were used to normalize protein loading.

### Calpain cleavage assays

In vitro cleavage of SYNJ1 by calpain was analysed as previously described [2, 51] in lysates of HEK 293 cells transfected with Flag-SYNJ1 145 kDa neuronal isoform with Lipofectamine 2000. Twenty-four hours after transfection, the cells were harvested in HEPES buffer (20 mM HEPES, pH 7.4, 1% Triton X-100, 100 mM KCl, 0.1 mM dithiothreitol, complete protease inhibitor tablet). Calpain cleavage was tested in the following conditions: Condition 1- incubation without adding CaCl_2_; Condition 2 – calpain was activated by adding 2 mM CaCl_2_ to the lysate; Condition 3 - calpain activation in the presence of 2 mM CaCl_2_ was inhibited by calcium chelators (2 mM EDTA, 2 mM EGTA); Condition 4 – calpain activation in the presence of 2 mM CaCl_2_ was blocked by pharmacological inhibitor (400 μM calpain inhibitor I A6185 from Sigma). Cell lysates were kept on ice for 30 min and were vortexed every 10 min for solubilisation. The lysates were then centrifuged at 16,000 x g for 15 min at 4 °C. The supernatant was incubated at 37 °C for 1 h in the 4 conditions detailed above. The reaction was stopped by adding Laemmli buffer followed by incubation at 100 °C for 10 min. The samples were analysed by WB.

### Statistical analysis

Statistical significance of comparisons was determined by unpaired Student’s *t-*tests, by *t*-test with Welch’s correction for unequal variances or by one-way ANOVA with *post-hoc* Tukey test using Prism 4 software (Graphpad).

## Results

### SYNJ1 is accumulated in neurons, plaque-associated dystrophic neurites and Hirano bodies in AD brains

**Fig. 1:**
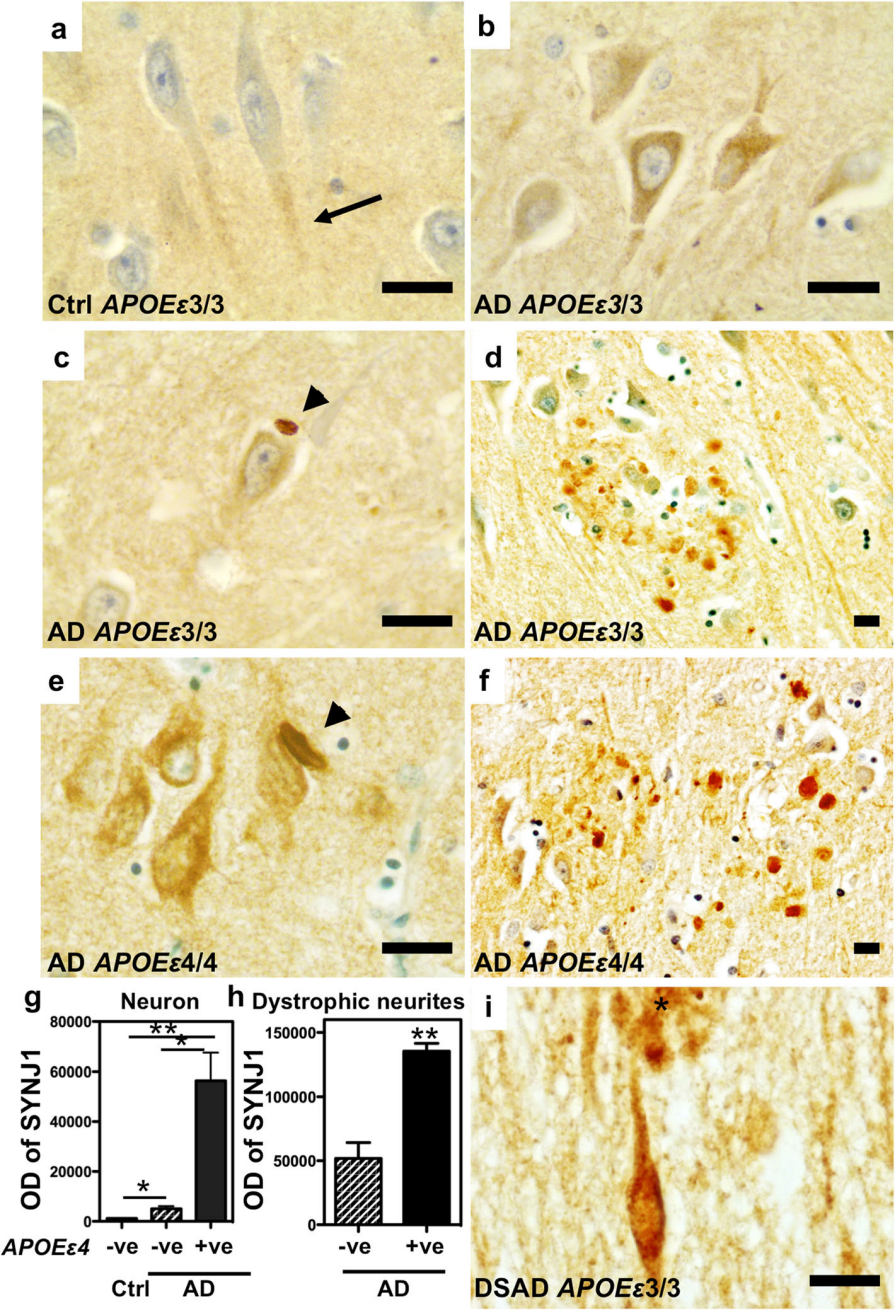
SYNJ1 is accumulated in neurons, Hirano bodies and plaque-associated dystrophic neurites in AD brains. **a** Immunostaining of SYNJ1 in hippocampal CA1–2 area of a control case carrying *APOEε*3/3. In control brains, SYNJ1 immunoreactivity was detected in dendrites in the hippocampal pyramidal neurons (**a**, arrow). **b**-**d** Immunostaining of SYNJ1 in hippocampal CA1–2 area of an AD patient carrying *APOEε*3/3. Neuronal perikarya and a perinuclear rim were immunostained for SYNJ1 (**b**). Ovoid intraneuronal inclusions similar to Hirano bodies were stained by anti-SYNJ1 antibody (**c**, arrowhead). Dystrophic neurites surrounding amyloid plaques were also stained for SYNJ1 (**d**). **e**-**f** In AD brains carrying *APOEε*4 allele(s), the intensity of SYNJ1 staining is stronger than in AD cases without *APOEε*4 allele (**e**). SYNJI positive Hirano bodies were also detected (**e**, arrowhead). A strong SYNJ1 immunoreactivity was observed in plaque-associated dystrophic neurites in CA1–2 area of AD cases carrying *APOEε*4 alleles (**f**). **g** Quantification of neuronal SYNJ1 immunoreactivity by image analysis in three groups: control without *APOEε*4 allele, AD group without *APOEε*4 allele and AD group carrying *APOEε*4 allele(s) (*n* = 3 for each group). Paraffin section of control cases bearing *APOEε*4 allele was not available and was not included in the analyses. SYNJ1 immunoreactivity is significantly increased in AD cases carrying *APOEε*4 alleles compared to AD cases without *APOEε*4 allele. *p < 0.05 and **p < 0.01 by one way ANOVA with *post-hoc* Tukey test. **h** Quantification of SYNJ1 immunoreactivity by image analysis in plaque-associated dystrophic neurites. SYNJ1 immunoreactivity is significantly increased in AD cases carrying *APOEε*4 alleles compared to AD cases without *APOEε*4 allele (*n* = 3 for each group). **p < 0.01 by unpaired *t-test*. **i**. In the hippocampus of DSAD brain, a strong immunostaining for SYNJ1 was detected in neuronal perikarya and in dystrophic neurites surrounding amyloid plaques (asterisk). *Scale bars* 10 μm

Five anti-SYNJ1 antibodies were characterized. Anti-SYNJ1 HPA011916 antibody provided specific signal in immunostaining and WB and thus was used throughout this study (Supplementary Fig. 1, online resource). Immunohistochemistry was performed using anti-SYNJ1 HPA011916 antibody on paraffin embedded hippocampal sections of human *post-mortem* brains from non-demented control, AD patients carrying no or *APOEε4* allele(s) and DSAD (Fig. 1). In control brains, SYNJ1 immunoreactivity was detected in the neuropil, corresponding to nerve terminals and in the cytoplasm of neurons as previously reported [6, 7, 39, 40, 60] (Fig. 1a). In pyramidal hippocampal neurons in AD, SYNJ1 staining was observed as puncta in neuronal perikarya and in dendrites, in a perinuclear rim and in neuronal processes (Fig. 1b). In pyramidal neurons of the CA1–2, the intensity of SYNJ1 labelling was increased in AD brains compared to control brains (Fig. 1a-c, e). Hirano bodies are often found in CA1 pyramidal neurons in AD brains and less frequently in non-demented aged brains [27]. Hirano bodies were strongly immunostained by anti-SYNJ1 antibody (Fig. 1c, e), as well as dystrophic neurites surrounding amyloid deposits (Fig. 1d, f). It has been reported that inefficient *SYNJ1* mRNA degradation is linked to *APOEε4* genotype in human brains and mouse models [61]. The intensity of SYNJ1 staining was increased in the neuronal soma and in plaque-associated dystrophic neurites in the CA1–2 areas of AD cases carrying one or two *APOEε4* allele(s) compared to those carrying no *APOEε4* allele (Fig. 1g, h). We counted in the latter areas the absolute number of Hirano bodies stained by anti-SYNJ1 antibody, but there was no statistically significant difference in AD with or without *APOEε4* allele due to a large degree of variability in our study cohort (data not shown). *SYNJ1* maps to chromosome 21 and SYNJ1 labelling is enhanced in neurons of DS brains [7]. We confirmed in DSAD a prominent SYNJ1 immunoreactivity in the somatodendritic compartment in neurons and in plaque-associated dystrophic neurites (Fig. 1i). These data suggest that SYNJ1 immunolabelling is increased in AD brains in association with *APOEε4* and subcellular localization of SYNJ1 is altered in AD brains.

### SYNJ1 is partially associated with hyperphosphorylated tau in NFTs of AD brains

**Fig. 2:**
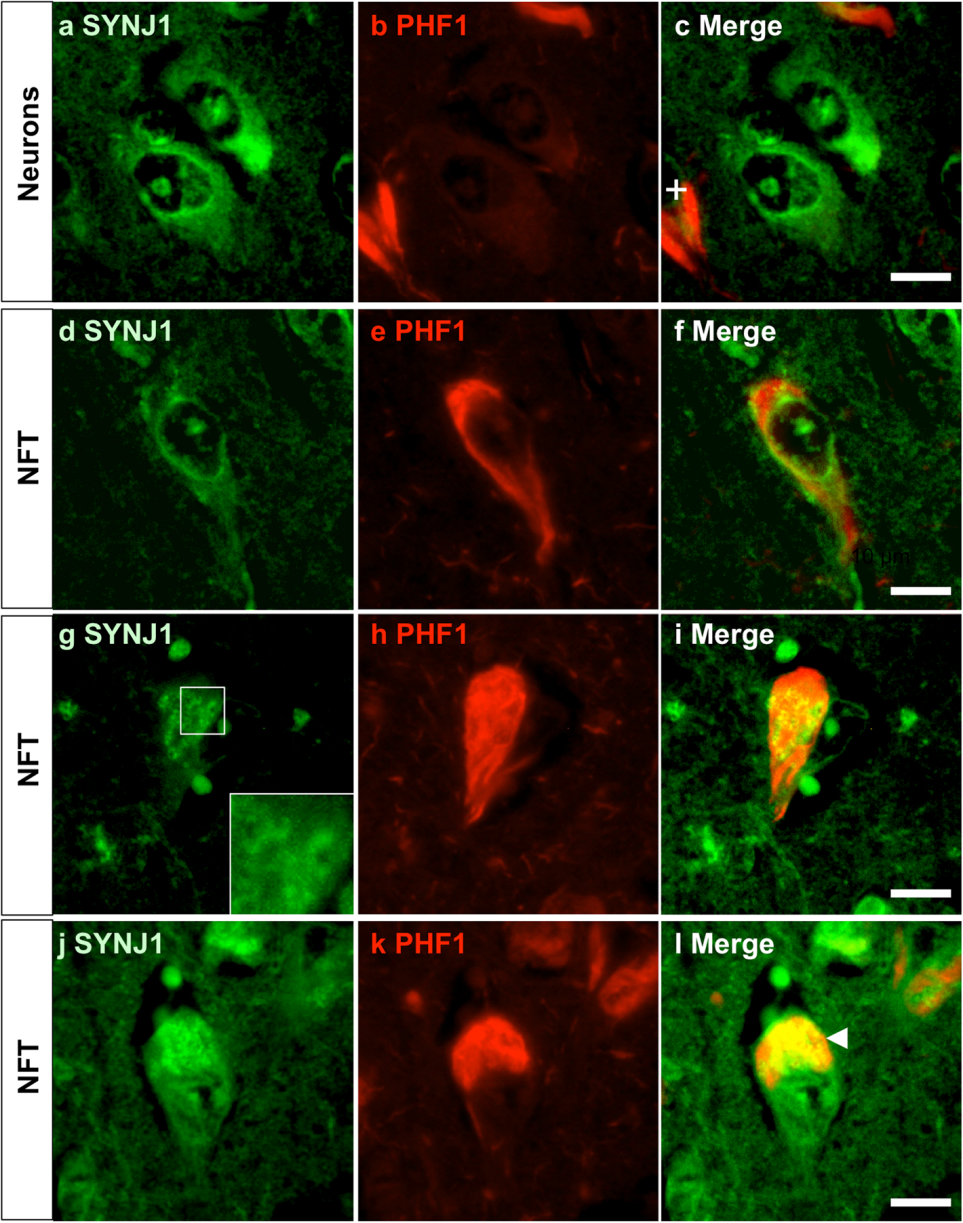
Detection of SYNJ1 immunoreactivities in intraneuronal granular structures in AD brains. **a**-**l** Double immunostaining for SYNJ1 (**a**, **d**, **g**, **j**, *green*) and PHF1 (**b**, **e**, **h**, **k**, *red*) shows that SYNJ1 immunoreactivity is detected as a perinuclear rim and in the perikarya in non-tangle bearing neurons (**a**-**c**) and in tangle bearing neurons (**d**-**l**) in the CA1–2 pyramidal neurons of AD hippocampus. SYNJ1-positive intraneuronal granular or donuts-like structures (**g**, inset) were occasionally detected in tangle bearing neurons and were surrounded and sometimes overlapped with hyperphosphorylated tau in NFTs (**l**, arrowhead). Representative images of an *APOEε*3/3 AD case are shown. *Scale bars* 10 μm

In order to confirm whether somatodendritic SYNJ1 immunoreactivity was associated with neuropathological lesions in the AD hippocampus, a double immunostaining was carried out using anti-SYNJ1 and PHF1 antibodies (Fig. 2a-l). PHF1 recognises tau doubly phosphorylated at Ser396 and Ser404 and labels NFTs and dystrophic neurites [8]. A somatodendritic SYNJ1 immunoreactivity was detected in non-tangle bearing neurons in AD brains (Fig. 2a-c). SYNJ1 positive structures with granular or donut-shaped forms were also detected and were surrounded or sometimes co-localized with hyperphosphorylated tau in the NFTs (Fig. 2d-l). In CA1 to CA2 region of the AD hippocampus, approximately half of all the NFTs in CA1–2 areas had SYNJ1-positive granular structures. On the other hand, some NFTs were almost devoid of SYNJ1 immunoreactivity (Fig. 2c cross).

### SYNJ1 is accumulated in Hirano bodies

**Fig. 3:**
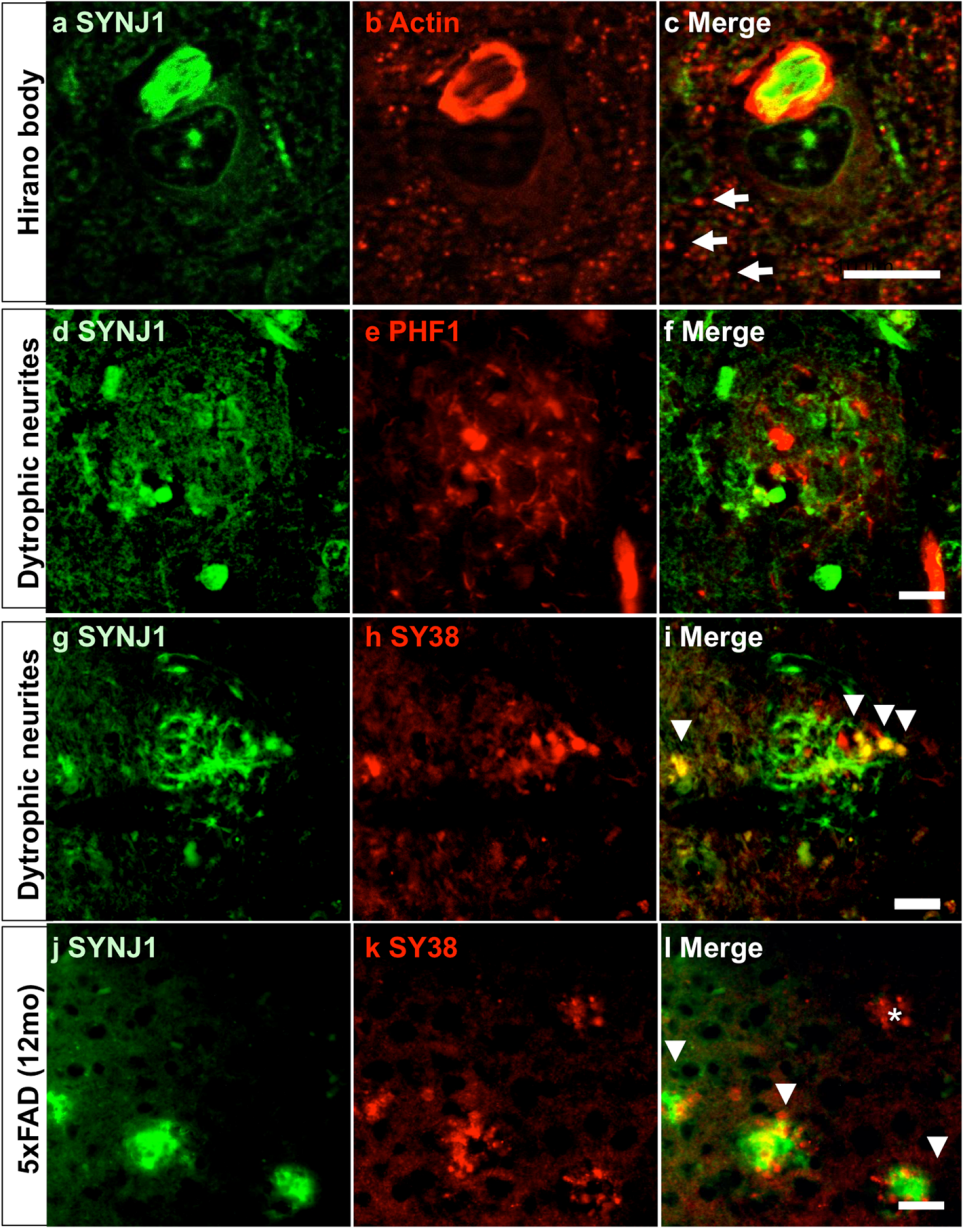
Detection of SYNJ1 immunoreactivities in Hirano bodies and in plaque-associated dystrophic neurites in AD brains. **a**-**c** A double immunofluorescence labelling for SYNJ1 (**a**, *green*) and actin (**b**, *red*) confirmed that Hirano bodies were immunostained for SYNJ1 in pyramidal neurons of AD brains (**c**, merge). While SYNJ1 labelling was stronger in the centre of the Hirano bodies (**a**), actin labelling was stronger in the periphery of Hirano bodies (**b**). There were numerous smaller punctiform structures that were actin positive but SYNJ1 negative in AD brains (arrows). **d**-**f** A double immunofluorescence labelling for SYNJ1 (**d**, *green*) and PHF1 (**e**, *red*) does not show association of SYNJ1-positive globular structures and hyperphosphorylated tau in the dystrophic neurites surrounding senile plaques. **g**-**l** A double immunofluorescence labelling for SYNJ1 (**g**, **j**, *green*) and Synaptophysin (SY38, **h**, **k**, *red*) shows partial colocalization of SYNJ1 and Synaptophysin (arrowheads) in AD brain (**g**-**i**) and in 5XFAD mouse brains at 12 months (**j**-**l**). Some plaque-associated dystrophic neurites were yet devoid of SYNJ1 immunoreactivity (**l**, asterisk). Representative images of an *APOEε*3/3 AD case (**a**-**i**) and 5XFAD (**j**-**l**) are shown. *Scale bar* 10 μm for **a**-**c** and 40 μm for **d**-**l**

There are two common actin-positive lesions in AD brains: Hirano bodies and much smaller ADF/cofilin rods [41]. To confirm the presence of SYNJ1 immunoreactivity in Hirano bodies, a double-immunofluorescence staining was carried out for SYNJ1 and actin. Actin positive Hirano bodies were systematically immunostained by anti-SYNJ1 antibody but with incomplete overlap: whereas the periphery of Hirano body was strongly stained by anti-actin antibody [36], the centre of Hirano body was strongly labelled by anti-SYNJ1 antibody (Fig. 3a-c). While much smaller actin-positive punctiform staining was remarkable in AD brains, such actin-positive punctiform structures were not immunostained with anti-SYNJ1 antibody (Fig. 3c arrows).

### SYNJ1 is partially colocalized with Synaptophysin in plaque-associated dystrophic neurites in AD and 5XFAD brains

A SYNJ1 immunoreactivity was detected as globular structures around amyloid plaques in AD brains (Fig. 1d, f). The great majority of SYNJ1 positive structures observed around the amyloid deposits were not directly colocalized with hyperphosphorylated tau (Fig. 3f). Synaptophysin is a presynaptic marker and is accumulated in the axons of plaque-associated dystrophic neurites in AD brains [13] and in 5XFAD mouse brains [52]. SYNJ1 was partially colocalized with Synaptophysin in the plaque-associated dystrophic neurites in AD brains (Fig. 3g-i) and in the 5XFAD mouse brain (Fig. 3j-l). Taken together, these data suggest that SYNJ1 is clearly associated with Alzheimer lesions such as NFTs, Hirano bodies and Synaptophysin-positive dystrophic neurites surrounding amyloid plaques.

### *SYNJ1* mRNA is increased in AD brains in association with *APOEε4* genotype

**Fig. 4:**
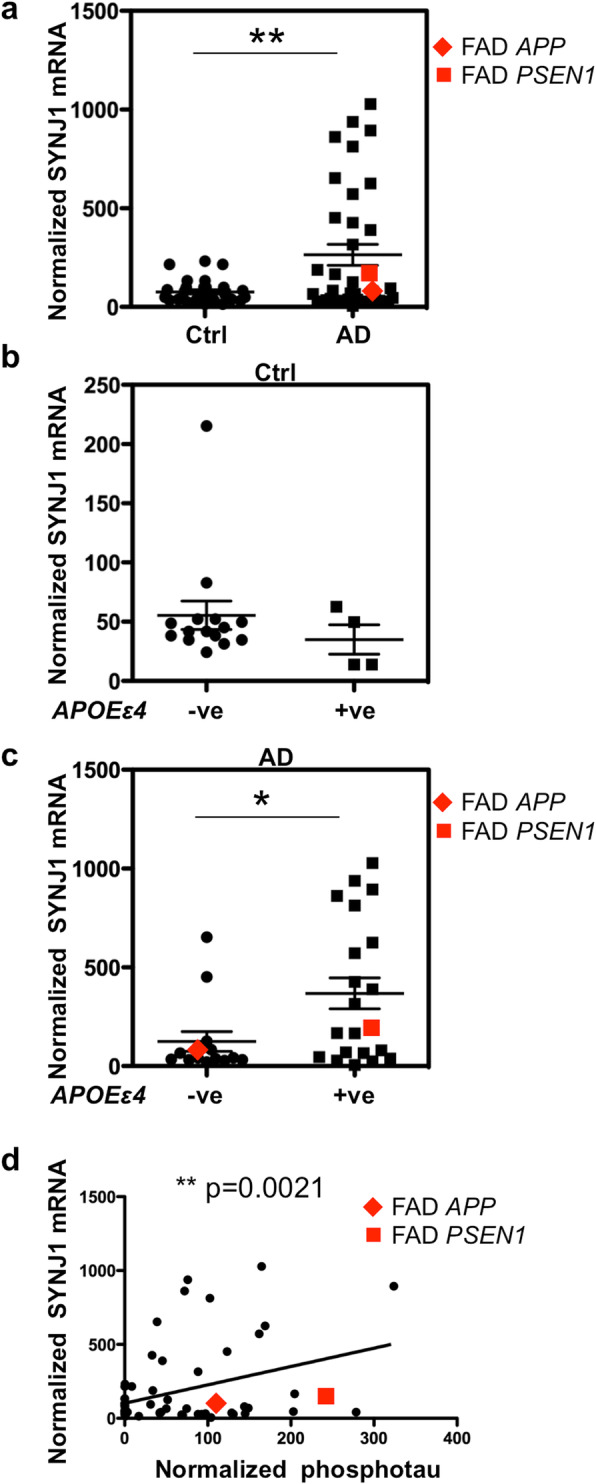
Expression of *SYNJ1* mRNA in human *post-mortem* AD brains. **a** qPCR for *SYNJ1* was performed on the mRNA extracted from control (*n* = 30) and AD (*n* = 36) T1 isocortex. The level of normalized *SYNJ1* mRNA was significantly increased in AD brains. Two FAD cases are shown in red. **b** No significant difference was observed in the normalised *SYNJ1* mRNA levels between non-carriers (*n* = 15) and carriers (*n* = 4) of *APOEε*4 allele(s) in the control cohort. **c** In AD cohorts, *SYNJ1* mRNA level was significantly higher in *APOEε*4 carriers (*n* = 21) than in non-carriers (*n* = 14). FAD cases are shown in red. *p < 0.05, **p < 0.01, by unequal variances *t* test. **d** There was a significant correlation between the levels of *SYNJ1* mRNA and phosphorylated tau detected by WB using PHF1. (r^2^ = 0.3713, *n* = 66, *p* = 0.0021, by Pearson correlation test)

In order to know whether the expression of SYNJ1 is altered in AD brains, *SYNJ1* mRNA level in T1 isocortex was analysed by qPCR in control and AD cases including 2 FAD cases with *APP* or *PSEN1* mutation (Supplementary Table 2, online resource). There was a significant increase of *SYNJ1* transcripts in AD brains compared to controls (Fig. 4a). *SYNJ1* transcripts of two FAD cases were in the 95% range and were not distinguishable from sporadic AD cases. To analyse a possible association of the *APOEε4* allele and *SYNJ1* mRNA level, *SYNJ1* transcript levels were compared between *APOEε4* non-carriers and *APOEε4* carriers (one or two alleles) of the control and AD cohorts (Fig. 4b-c). There was a significant increase of *SYNJ1* mRNA levels in the *APOEε4* carriers compared to non- *APOEε4* carriers in AD group (Fig. 4c). Such *APOEε4-*dependent increase of *SYNJ1* mRNA level was not observed in the control group in our study cohort (Fig. 4b). There was a significant correlation between the level of *SYNJ1* mRNA and hyperphosphorylated tau detected with PHF1 antibody by WB (Fig. 4d). These data indicate that there was a significant increase of *SYNJ1* transcripts in AD brains compared to control and that this increase was associated with *APOEε4* genotype and tau load in AD cohorts.

### SYNJ1 protein becomes highly insoluble and is detected in sarkosyl-insoluble fraction in AD brains

**Fig. 5:**
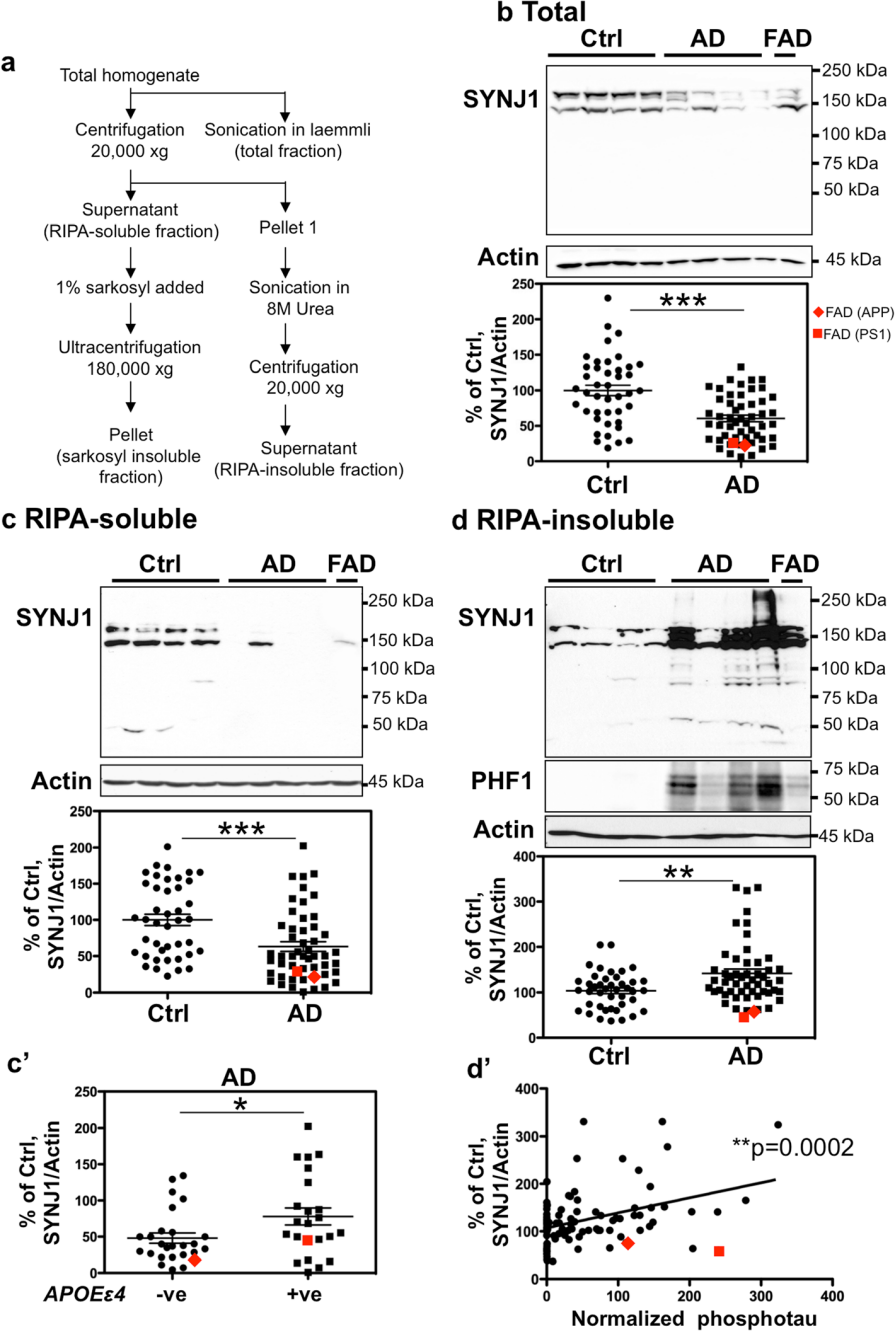
Insoluble SYNJ1 is increased in AD brains and is correlated to tau load. **a** Summary of the fractionation protocol used to obtain total, RIPA-soluble, RIPA-insoluble and sarkosyl-insoluble fractions. **b** SYNJ1 was significantly decreased in the total homogenate of AD T1 isocortex. **c** SYNJ1 was significantly decreased in RIPA-soluble fraction of AD brains. **c’** There was a significantly higher level of SYNJ1 protein detected in the AD cases carrying *APOEε*4 allele(s) compared to the AD cases without *APOEε*4 allele. **d** SYNJ1 was increased in RIPA-insoluble fraction of AD brains. Lower MW SYNJ1 positive bands were detected approximately around 100 kDa, 80 kDa and 50 kDa in the AD brain. For **b**-**d**, T1 isocortex from control (*n* = 42) and AD (*n* = 50) including 2 FAD cases with *APP* or *PSEN1* mutation were analysed. **p < 0.01, ***p < 0.001, by unequal variances *t* test. **d’** There was a significant positive correlation between the levels of SYNJ1 protein and phosphorylated tau detected using PHF1 in the RIPA-insoluble fraction. (r^2^ = 0.1443, *n* = 92, *p* = 0.0002, by Pearson correlation test)

**Fig. 6:**
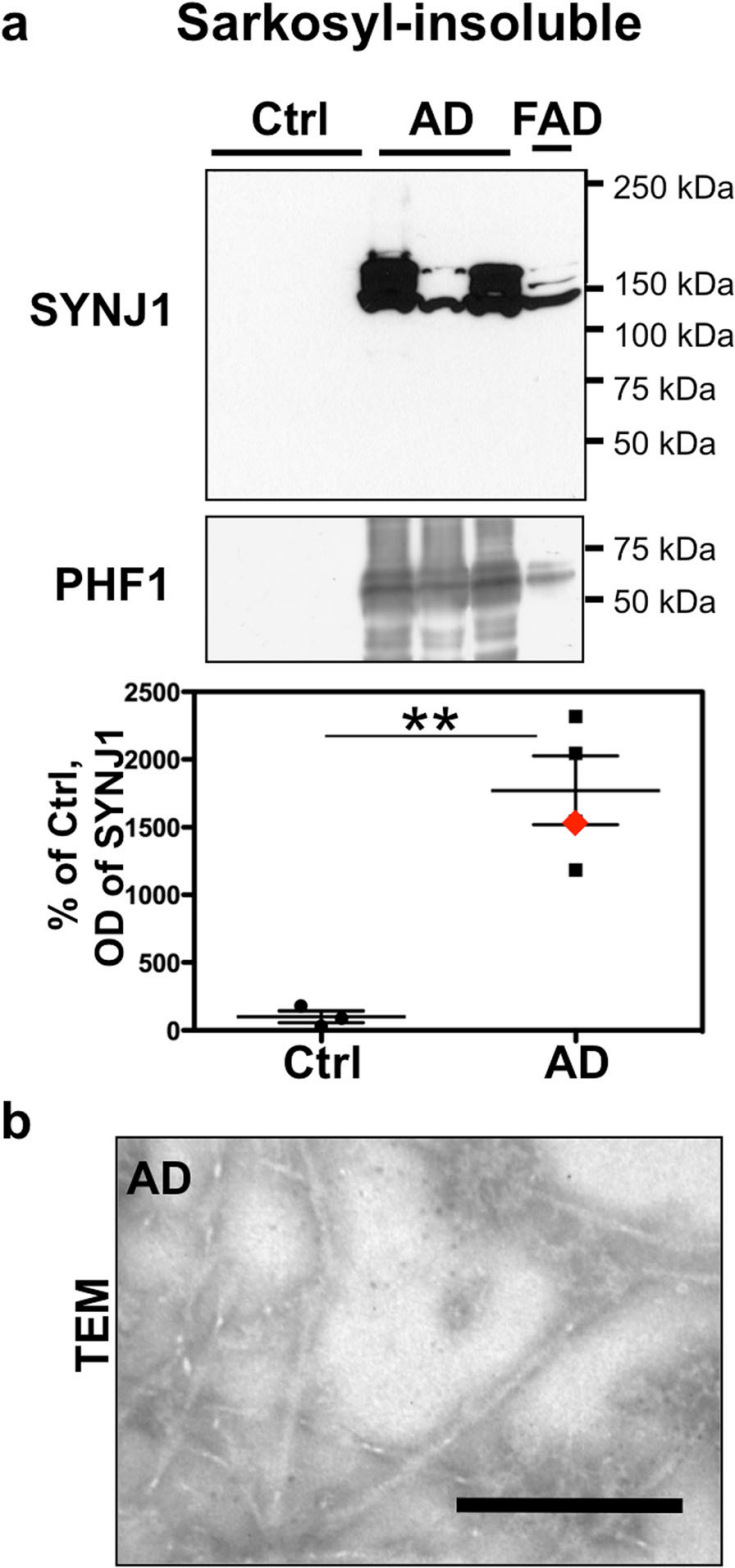
SYNJ1 is co-enriched with PHF-tau in sarkosyl-insoluble fraction. **a** WB for SYNJ1 and PHF1 in sarkosyl-insoluble fraction of control (*n* = 3) and AD (SAD *n* = 3, FAD with *APP* mutation *n* = 1). SYNJ1 was enriched in sarkosyl-insoluble fractions with PHF-tau. **b** Representative image of the sarkosyl-insoluble PHF-tau of a sporadic AD case taken by transmission electron microscopy. *Scale bar* 0.2 μm

We subsequently analysed SYNJ1 protein expression by WB in brain lysates of T1 isocortex (Fig. 5). First, brain homogenates were sonicated on ice in the presence of Laemmli sample buffer and analysed by WB. In spite of the increased level of *SYNJ1* mRNA, normalized SYNJ1 protein level (145 and 170 kDa isoforms) was significantly decreased in total fraction of AD brains (Fig. 5b). We wondered whether SYNJ1 might become highly insoluble in AD brains and might not be completely detectable in our homogenisation protocol. Fractionation and urea solubilisation were carried out in order to verify potential changes in SYNJ1 partitioning between soluble and insoluble fractions and to better solubilize insoluble proteins (Fig. 5c-d). The level of SYNJ1 in the RIPA-soluble fraction was decreased in AD brains (Fig. 5c). SYNJ1 protein was increased in the AD cases carrying one or two *APOEε4* allele(s) compared to AD cases carrying no *APOEε4* allele (Fig. 5c’). On the contrary to the RIPA-soluble fraction, the level of SYNJ1 protein detected in the RIPA-insoluble fraction containing largest protein aggregates was significantly increased in AD brains (Fig. 5d). There were some cleaved bands of SYNJ1 clearly detected in AD brains in the RIPA-insoluble fraction approximately at 50, 80 and 100 kDa (Fig. 5d). Cleaved fragments of SYNJ1 were less frequently observed in the RIPA-insoluble fraction of control brains. Some AD cases exhibiting severe tau load contained SYNJ1 positive smears at higher molecular weight than 145 and 170 kDa in RIPA-insoluble fraction (Fig. 5d). There was a significant correlation between the levels of SYNJ1 and PHF1-positive hyperphosphorylated tau in the RIPA-insoluble fraction (Fig. 5d’). To determine whether enrichment of SYNJ1 in insoluble fraction was related to interaction with PHF-tau, sarkosyl fractionation was carried out to analyse PHF-tau fraction [14, 26]. WB analysis of sarkosyl-insoluble fraction suggested that SYNJ1 was co-precipitated in the sarkosyl-insoluble fraction of AD brains (Fig. 6a) where PHF-tau was enriched (Fig. 6b). These data suggest that SYNJ1 undergoes a significant solubility change in AD brains and is detected in the PHF-tau fraction.

### SYNJ1 is a substrate of calpain

**Fig. 7:**
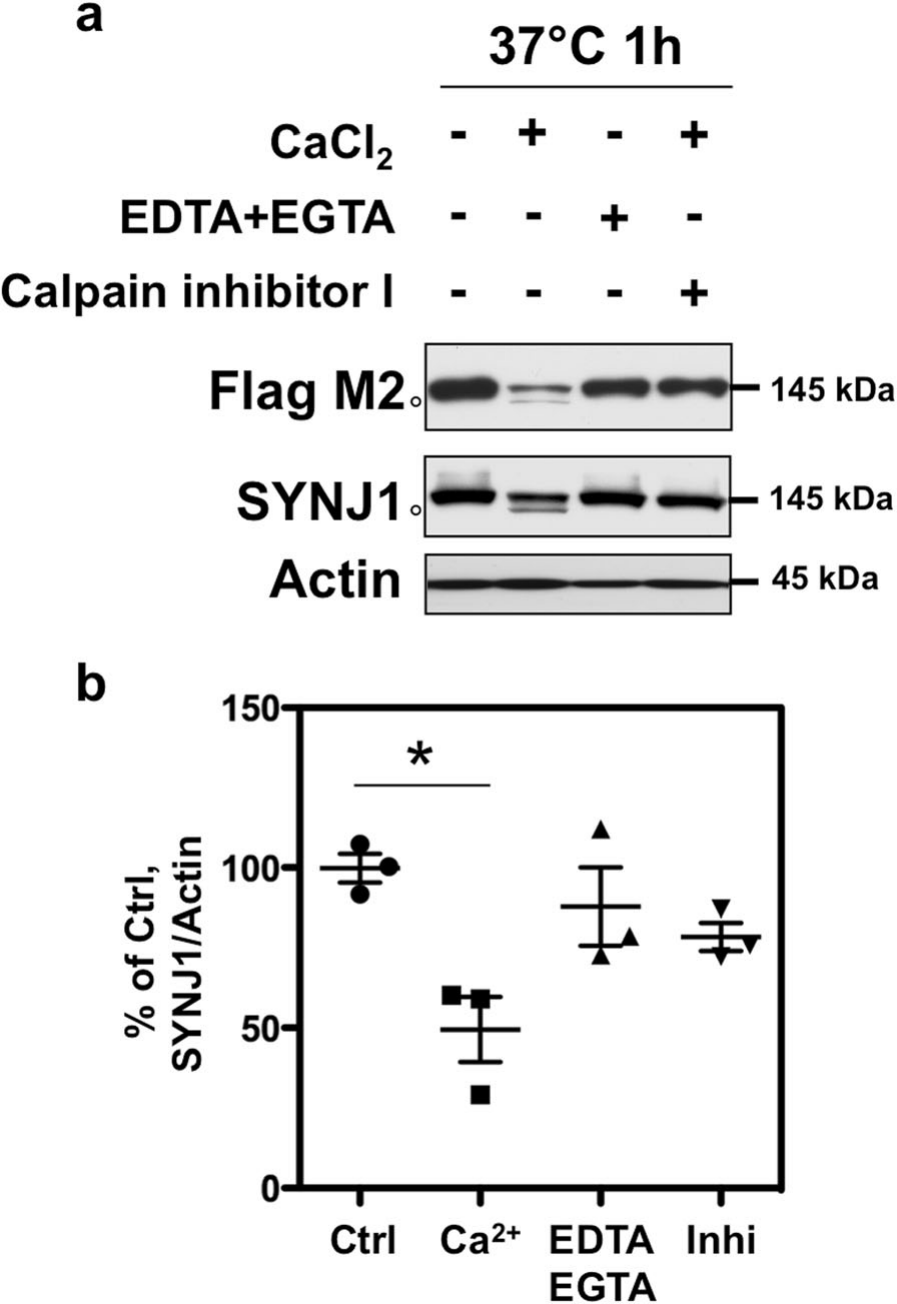
In vitro cleavage assay for SYNJ1 by calpain. **a** HEK 293 cells were transiently transfected with Flag-SYNJ1 145 and cultured for 24 h. The cell lysate was incubated at 37 °C for 1 h in the presence or absence of calcium. In the presence of calcium in the lysate, SYNJ1 was significantly decreased by 50% and a 140-kDa band appeared (open circle). The proteolysis was inhibited by adding calcium chelators (EDTA and EGTA) or calpain inhibitor I. **b** The graph shows the OD of SYNJ1 normalised to actin in each condition of three independent experiments. *p < 0.05 by one-way ANOVA with *post-hoc* Tukey test

There were several bands below 145 kDa that were detected by SYNJ1 antibody in the RIPA-insoluble fraction of AD brains (Fig. 5c). Such SYNJ1-positive bands might result from proteolytic cleavage by proteases such as calpain abnormally activated in AD brains [31, 53]. To test whether SYNJ1 is proteolysed by calpain, HEK 293 cells were transfected with a plasmid encoding SYNJ1 145 Flag-tagged at its N terminus, and in vitro cleavage assays were carried out as previously described [2, 51]. The cells were lysed in HEPES buffer and incubated at 37 °C in the presence or absence of Ca^2+^. After 1 h incubation at 37 °C in the presence of Ca^2+^, SYNJ1 protein was decreased by 50% and a 140 kDa-band below the main 145 kDa-band appeared (Fig. 7a-b). The band observed at 140 kDa was detected by both anti-Flag M2 (N-terminus) and HPA011916 (detecting 962–1092 amino acids of human SYNJ1) antibodies, and thus was considered as SYNJ1 cleaved at its extreme C-terminus in this condition. The SYNJ1 cleavage was partially inhibited by adding Ca^2+^ chelators (EDTA and EGTA) or a calpain inhibitor (Fig. 7). These data suggest that calpain activation leads to a significant cleavage of SYNJ1 and may be involved in SYNJ1 reduction in the total fraction and also in producing SYNJ1 proteolytic fragments observed in the RIPA-insoluble fraction of AD brains.

## Discussion

In the present study, we assessed the changes in SYNJ1 localization and expression level in *post-mortem* AD brain tissues. By immunohistochemistry, we determined that SYNJ1 accumulates in the neuronal soma and in plaque-associated dystrophic neurites and is associated to Hirano bodies. SYNJ1 immunoreactivity occasionally colocalized with hyperphosphorylated tau in NFTs and Synaptophysin positive dystrophic neurites. SYNJ1 immunoreactivity in neurons, *SYNJ1* transcripts and insoluble SYNJ1 proteins were significantly increased in AD cases and higher in AD patients bearing *APOEε*4 allele(s) compared to those bearing no *APOEε*4 allele. By biochemical fractionation, we provide evidence that SYNJ1 was co-enriched with PHF-tau in sarkosyl-insoluble fraction in AD brains. SYNJ1 was predominantly detected in the insoluble fraction in AD brains and several cleaved SYNJ1 fragments were also observed in the RIPA-insoluble fraction. Results from in vitro cleavage assays showed that SYNJ1 is a proteolytic substrate for calpain, which is activated in AD brains. These results constantly support our hypothesis that SYNJ1 is significantly misregulated in AD brains.

### SYNJ1 is associated with Hirano bodies, NFTs and dystrophic neurites in AD brains

Hirano bodies, eosinophilic crystal-like structures, are frequently observed in pyramidal neurons of the CA1 area of the Ammon’s horn of AD brains, other neurodegenerative diseases and during aging [27]. Hirano bodies are composed of intracellular aggregates of actin and actin-binding proteins [24, 35]. We observed that SYNJ1 was partially colocalized with actin, which is highly accumulated in Hirano bodies. SYNJ1 is involved in actin organisation in the cell via regulation of PIP_2_ that binds to actin-regulating proteins [54]. The proline-rich domain of SYNJ1 binds to SH3 domains of several actin regulating proteins such as Myosin 1E [30] or DAP160/intersectin [28]. The presence of the latter proteins in Hirano bodies has not been documented to our knowledge and further analyses are necessary to uncover the roles of SYNJ1 and its binding-partners in Hirano body formation. Reduction of soluble SYNJI and its proteolysis by calpain may lead to its dysfunction and may be responsible for a disorganization of the actin network leading to the formation of Hirano bodies. Actin positive rod shaped structures can be induced in cultured hippocampal neurons by oxidative stress or Aß treatment [41], and are also observed in tau transgenic rTg4510 mouse brains [23] and in APP transgenic Tg2576 mouse brains [37]. How these small actin positive rods are related to Hirano body formation remains largely unclear. Our data show that SYNJ1 is associated only to Hirano bodies but not clearly to other actin-positive structures, suggesting that the association of SYNJI with Hirano bodies is specific.

PIP_2_ is a substrate of SYNJ1 and plays important roles in many cellular signalling pathways [21]. PIP_2_ is accumulated and enriched in NFTs and granulovacuolar degeneration [45] and is colocalized with tau kinases such as CDK5 or GSK3ß [44]. Immunolabelling of SYNJ1 positive granular or donut-like structures were clearly observed in perinuclear rim and perikarya of NFTs of AD brains. A molecular interaction between SYNJ1 and PHF1-positive phosphotau are supported by our results of partial colocalization in NFTs, co-enrichment in sarkosyl-insoluble fraction and a significant correlation between SYNJ1 and phophotau in RIPA insoluble fraction.

SYNJ1 was also accumulated in dystrophic neurites around amyloid deposits. Presynaptic terminal swellings can be found in the corona of the senile plaques, where Synaptophysin [13], APP [11] or ADF/cofilin accumulate [41]. Taken together, we report for the first time a close association of AD neuropathological lesions and SYNJ1 in *post-mortem* AD brains.

### SYNJ1 is enriched in the insoluble fraction of AD brains

Despite the increase of *SYNJ1* mRNA, SYNJ1 protein level in the total and RIPA-soluble fractions was decreased in AD brains. Our observation on the reduction of SYNJ1 in the total and RIPA soluble fractions is consistent with a previous study reporting a decrease of SYNJ1 protein level in the 1% SDS soluble fraction of AD brain lysates [38]. The reduction of SYNJ1 in soluble fraction might be caused by multiple mechanisms of post-transcriptional and/or post-translational modifications of SYNJ1. Firstly, SYNJ1 may be sequestered by insoluble PHF-tau and may be trapped into NFTs. This hypothesis is supported by our results on co-enrichment of PHF-tau and SYNJ1 in sarkosyl-insoluble fraction and partial co-localization of SYNJ1 and hyperphosphorylated tau in NFTs. The smear-like migration pattern of SYNJ1 on SDS-PAGE that we observed in RIPA-insoluble fraction was quite similar to that of aggregate-prone proteins such as tau or TDP-43 [43]. Secondly, post-translational modifications (e.g. proteolysis, phosphorylation, oxidation etc.) of SYNJ1 may further modify SYNJ1 solubility. We demonstrated that SYNJ1 is a substrate of calpain, a protease highly activated in AD brains [53]. A number of neurotoxic factors, including Aß, can activate calpain [32]. Calpain may be involved in the reduction of SYNJ1 in the total fraction of AD and may also be involved in the formation of cleaved fragments of SYNJ1 detected in the RIPA-insoluble fractions of AD brains. We cannot exclude the possibility that other proteases may as well be involved in SYNJ1 cleavage. Further analyses on post-translational modification including phosphorylation or oxidation of SYNJ1 are necessary to decipher other potential mechanisms underlying solubility change of SYNJ1 observed in AD brains.

### SYNJ1 and *APOEε4* in AD brains

Our study provides the first evidence that SYNJ1 immunolabelling in neurons or in plaque-associated dystrophic neurites was increased in AD cases carrying one or two *APOEε4* alleles compared to those carrying no *APOEε4* allele. *SYNJ1* transcripts were significantly increased in AD brains compared to age-matched controls. Increase of *SYNJ1* mRNA in *APOEε4* carriers in our AD cohort is consistent with the previously reported upregulation of *SYNJ1* mRNA in *APOEε4* knock-in mouse brains and human *APOEε4* carriers [61], supporting the hypothesis that SYNJ1 is involved in the *APOE*-related AD susceptibility.

### SYNJ1 misregulation and AD

Our data shows that SYNJ1 undergoes significant alterations in expression, solubility and subcellular localization in AD. Increased level of insoluble SYNJ1 in AD brains may induce toxicity and could be associated with synaptic dysfunction and eventually with cognitive deficits as previously reported [59, 61]. Misregulation of SYNJ1 in AD may contribute to excitotoxicity [49], dysregulation of endocytosis [17] and deficits in autophagy [5, 46], all defects reported in AD patients and models.

Reduction of SYNJ1 has been proposed as a therapeutic target for amyloid-induced toxicity [10], amyloid clearance [62] and tau pathology after traumatic brain injury [15]. Nevertheless, a complete or partial loss of function of SYNJI protein may lead to profound pathological effects. Synj1-deficient mice die shortly after birth [20] and a homozygous truncating mutation within *SYNJ1* resulted in a neurodegenerative tauopathy associated to a severe reduction in SYNJ1 protein level in human [22]. In addition, recent studies on knock-in models of an early-onset PD mutation of *SYNJ1* (R258Q) suggested critical roles of SYNJ1 for endocytic protein dynamics and for balancing excitatory and inhibitory transmission [16] as well as autophagosome maturation at presynaptic terminals [57]. Our present study provides strong evidence that SYNJ1 is misregulated in AD brains and points to the possibility that targeting abnormal modifications of SYNJ1 could open up new windows to novel therapeutic strategies against AD.

## Conclusions

These data strongly support the hypothesis that SYNJ1 undergoes significant alterations in its localization and solubility in AD brains.

## Supplementary information

**Additional file 1.**

## Data Availability

Data generated during this study are included in this published article and its supplementary information files.
