# Supplementary material for: The lipid phosphatase Synaptojanin 1 undergoes a significant alteration in expression and solubility and is associated with brain lesions in Alzheimer’s disease
Source: Acta Neuropathol Commun. 2020 Jun 3;8:79. doi: 10.1186/s40478-020-00954-1 (PMC7268631; doi:10.1186/s40478-020-00954-1)
Supplement: Supplementary file 1 — Additional file 1. [file 40478_2020_954_MOESM1_ESM.pdf]

Supplementary materials

The lipid phosphatase Synaptojanin 1 undergoes a significant alteration in expression and solubility and is associated with brain lesions in Alzheimer's disease

Kunie Ando[1, 2, 3], Marième Ndjim[3], Sabrina Turbant[2, 3], Gaëlle Fontaine[3], Gustavo Pregoni[3], Luce Dauphinot[3], Zehra Yilmaz[1], Valérie Suain[1], Salwa Mansour[1], Michèle Authelet[1], Robert De Dekker[1], Karelle Leroy[1], Benoît Delatour[3], Brain Bank NeuroCEB Neuropathology Network, Charles Duyckaerts[2, 3], Marie-Claude Potier*[3], Jean-Pierre Brion*[1]

[1]Laboratory of Histology, Neuroanatomy and Neuropathology, ULB Neuroscience Institute, Université Libre de Bruxelles (ULB), B-1070 Brussels, Belgium.

[2]Laboratoire de Neuropathologie Escourolle, Hôpital de la Pitié-Salpêtrière, AP-HP, Paris, France

[3]ICM Institut du Cerveau, CNRS UMR7225, INSERM U1127, UPMC, Hôpital de la Pitié-Salpêtrière, Paris, France

1 Supplementary Figure

2 Supplementary tables

***: Authors contributed equally**

Abbreviated title: **The expression and localization of SYNJ1 in Alzheimer brains.**

Send correspondence to:

Dr. Jean-Pierre Brion and Dr. Kunie Ando

Laboratory of Histology, Neuroanatomy and Neuropathology (CP 620)

ULB Neuroscience Insitute (UNI)

Université Libre de Bruxelles, Faculty of Medicine

808, route de Lennik (Bldg G)

1070 Brussels, Belgium

Office: +32 2 555 6333

Fax: +32 2 555 6285

jpbrion@ulb.ac.be and Kunie.Ando@ulb.ac.be

Characterization and selection of anti-SYNJ1 antibody to study post-mortem human brains

In order to investigate the localization and the expression level of SYNJ1 protein in human *post-mortem* tissues of Alzheimer disease (AD), we needed a specific anti-SYNJ1 antibody to detect human SYNJ1 protein by WB and immunohistochemistry. Five commercially available anti-SYNJ1 antibodies were tested in this study (Table S1). These antibodies generally target C-terminus of human SYNJ1 (HPA011916) or rat Synj1 (BD612249, sc-32770, ab19904, TA309245) (Fig. S1a). We first tested these antibodies by WB to detect human SYNJ1 protein in the brain lysate from a control non-demented human post-mortem brain lysate. Bands of SYNJ1 corresponding at 145 and 170 kDa were obtained using HPA011916, BD612249 but the others were not specific or did not recognize human SYNJ1 in our condition (Fig. S1b). We tested the validity of these two anti-SYNJ1 antibodies for immunohistochemistry on paraffin-embedded E17 embryonic rat brains where Synj1 is highly expressed [3]. HPA011916 labelled expected Synj1 staining in paraffin embedded sections of E17 rat brain cortex (Fig. S1c) while BD612249 did not (Fig. S1d). Consistently, BD612249 did not provide specific staining by immunohistochemistry on human *post-mortem* hippocampus of paraffin sections of (Fig. S1e) or cryosections (data not shown). The specificity of HPA011916 was further confirmed by immunocytochemistry in HEK 293 cells transfected with flag-tagged human SYNJ1 cDNA short isoform (145 kDa) as previously described [2]. Double-fluorescent staining for rabbit polyclonal anti-SYNJ1 HPA011916 antibody and mouse monoclonal anti-Flag M2 antibody showed a perfect co-localisation and provided evidence of the specificity of HPA011916 (Fig. S1f-g). HPA011916 was therefore considered to be specific and valid to detect SYNJ1 protein by both WB and by immunohistochemistry and thus used throughout this study.

Table 1

Cat. No.	Source	Epitope	Host	WB for human SYNJ1	IHC for human SYNJ1
HPA011916	SIGMA	Human SYNJ1 962-1092aa	Rabbit pAb	1/2000	1/100
BD612249	BD transduction	Rat Synaptojanin 1 1145-1259aa	Mouse mAb	1/2000	NG
sc-32770	SCBT	Rat Synaptojanin 1156-1286aa	Mouse mAb	NG	NG
ab19904	Abcam	Rat Synaptojanin 1250-1350aa	Rabbit pAb	NG (Non-specific bands)	NG
TA309245	Origene	Rat Synaptojanin 1 C-terminal region	Mouse mAb	NG (Rat Synj1 specific)	NG

Table S1. Anti-human SYNJ1 or anti-rat Synj1 antibodies tested in this study.

Five commercially available anti-human SYNJ1 or anti-rat Synj1 antibodies were tested for WB and IHC. NG: not good.

Figure S1

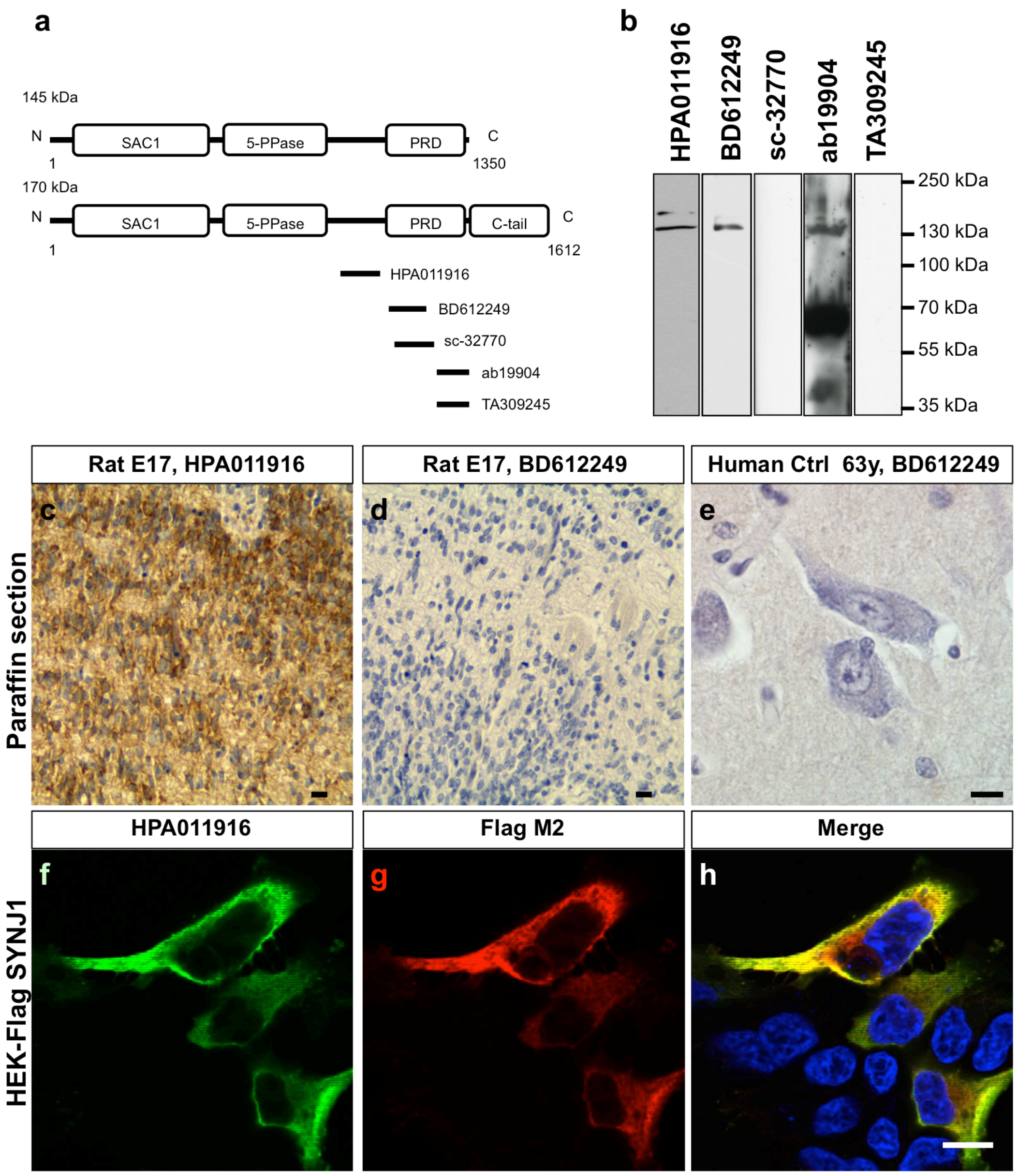

Supplementary Figure 1

Comparison of commercially available anti-SYNJ1 antibodies

a Schematic representation of SYNJ1 isoforms of 145 and 170 kDa and epitope of anti-human SYNJ1 or anti-rat Synj1 antibodies used in this study. Suppressor of actin (SAC1). 5' phosphatase (5-PPase). Proline-rich domain (PRD) of SYNJ1. **b** WB on the RIPA-soluble fraction from a human control T1 isocortex using anti-SYNJ1 antibodies. **c, d Representative photos of the subventricular zone of p**araffin-embedded rat brain sections of Embryonic day 17 (E17) immunostained using HPA011916 (**c**) and BD612249 (**d**) antibodies for Synj1. HPA011916 stained astroglia and neuronal precursors while BD612249 did not. **e** BD612249 did not detected specific signal in paraffin sections of human control hippocampus. **f-h** HEK 293 cells were transfected with Flag-conjugated SYNJ1 145 kDa isoform. After fixation, the cells were immunostained using anti-SYNJ1 HPA011916 antibody (**f**, *green*) and anti-Flag M2 antibody (**g**, *red*), merged with DAPI counterstaining (**h**, *blue*). Scale bars 10 µm.

Supplementary Table 2

Case #	Clinical diagnostics	Braak	Thal	Sex	Age	PMD	*ApoE*	Analyses	*SYNJ1* mRNA
No. 1	Control	III	4	F	82	NA	E3/E3	WB, QPCR	215,32
No. 2	Control	0	0	M	69	6	E2/E3	WB, QPCR	45,15
No. 3	Control	II	0	M	79	NA	E2/E3	WB, QPCR	24,31
No. 4	Control	0	0	F	60	28	E3/E3	WB, QPCR	34,73
No. 5	Control	0	NA	F	86	NA	NA	WB, QPCR	86,82
No. 6	Control	II	0	M	73	10	E3/E3	WB, QPCR	41,68
No. 7	Control	0	1	M	84	13.5	E3/E3	WB, QPCR	38,20
No. 8	Control	I	0	F	92	NA	NA	WB	99,38
No. 9	Control	III	0	M	58	5.5	NA	WB	34,73
No. 10	Control	III	NA	M	70	31	E3/E3	WB, QPCR	38,20
No. 11	Control	IV	2	F	76	28	E3/E3	WB, QPCR	41,68
No. 12	Control	IV	1	M	84	48	E3/E3	WB, QPCR	52,09
No. 13	Control	NA	NA	M	78	23	E3/E3	WB	
No. 14	Control	0	NA	M	72	24	E3/E3	WB, QPCR, IHC	31,26
No. 15	Control	0-I	NA	M	81	16,5	E3/E3	WB, QPCR, IHC	48,62
No. 16	Control	I	NA	F	89	35	E2/E4	WB, QPCR	62,51
No. 17	Control	0-I	NA	F	71	7	E3/E4	WB, QPCR	13,89
No. 18	Control	I-II	0	M	67	24	E3/E3	WB, QPCR	34,73
No. 19	Control	0-I	NA	M	73	9.5	E3/E4	WB	49,69
No. 20	Control	III	4	F	77	48	E3/E4	WB, QPCR	13,89
No. 21	Control	III	2	M	71	24	E3/E4	WB	
No. 22	Control	III	3	M	69	24	E3/E4	WB	
No. 23	Control	II	1	M	82	63	NA	WB, QPCR	132,51
No. 24	Control	IV	NA	M	92	NA	E3/E3	WB, QPCR	82,82
No. 25	Control	NA	NA	M	71	NA	E3/E3	WB, QPCR	49,69
No. 26	Control	NA	NA	M	80	4	NA	WB, QPCR	231,88
No. 27	Control	NA	NA	F	89	12	NA	WB, QPCR	82,82
No. 28	Control	NA	NA	F	80	NA	NA	WB	
No. 29	Control	NA	NA	M	66	NA	NA	WB	
No. 30	Control	III	NA	F	65	NA	NA	WB, QPCR	99,38
No. 31	Control	NA	NA	M	85	NA	NA	WB	
No. 32	Control	II	3	M	85	10	NA	WB	
No. 33	Control	NA	0	F	52	29	NA	WB, QPCR	99,38
No. 34	Control	II	NA	F	83	21	NA	WB, QPCR	215,32
No. 35	Control	III	0	F	92	NA	NA	WB	
No. 36	Control	0	NA	M	82	28	NA	WB	
No. 37	Control	IV	0	F	90	31	NA	WB, QPCR	99,38
No. 38	Control	III	5	F	94	NA	NA	WB	
No. 39	Control	NA	NA	F	66	NA	NA	WB, QPCR	132,51
No. 40	Control	III	5	M	81	NA	NA	WB	

No. 41	Control	III	5	M	76	27	NA	WB	
No. 42	Control	NA	NA	M	66	7	E2/E3	QPCR	52,09
No. 43	Control	0	0	M	63	NA	NA	IHC	
No. 44	AD	VI	4	M	60	37	E3/E3	WB, QPCR	652,64
No. 45	AD	VI	NA	M	67	19	E3/E4	WB	
No. 46	AD	VI	NA	M	57	19	E3/E4	WB, QPCR	165,63
No. 47	AD	VI	4	M	79	28	E3/E4	WB, QPCR	5,79
No. 48	AD	VI	NA	M	63	NA	NA	WB	
No. 49	AD	VI	NA	M	74	10	E3/E3	WB	
No. 50	AD	VI	NA	M	64	3	E3/E3	WB, QPCR	65,99
No. 51	AD	VI	NA	F	89	10	E2/E3	WB, QPCR	93,77
No. 52	AD	VI	NA	F	87	23	E2/E3	WB	
No. 53	AD	VI	5	F	86	30	E3/E3	WB, QPCR	41,68
No. 54	AD	VI	NA	M	71	6	E3/E3	WB, QPCR	451,48
No. 55	AD	VI	NA	F	90	32	E2/E4	WB, QPCR	388,97
No. 56	AD	VI	3	M	61	24	E3/E4	WB, QPCR	1027,98
No. 57	AD	VI	4	F	79	24	E3/E4	WB, QPCR	79,88
No. 58	AD	VI	5	M	82	25	E3/E4	WB, QPCR	27,78
No. 59	AD	VI	5	F	83	24.5	E3/E4	WB, QPCR	65,99
No. 60	AD	VI	4	M	83	21	E3/E4	WB, QPCR	316,04
No. 61	AD	VI	NA	M	76	9.5	E3/E4	WB, QPCR	24,31
No. 62	AD	VI	4	F	80	24	E3/E4	WB, QPCR	861,28
No. 63	AD	VI	NA	M	66	9.5	NA	WB, QPCR	27,78
No. 64	AD	VI	NA	M	81	20	E2/E3	WB	
No. 65	AD	VI	NA	F	82	20.5	E3/E3	WB, QPCR	31,26
No. 66	AD	VI	5	M	84	NA	E3/E3	WB, QPCR	125,03
No. 67	AD	VI	NA	F	91	26	E3/E3	WB, QPCR	37,07
No. 68	AD	VI	5	F	83	24	E3/E3	WB, QPCR	27,78
No. 69	AD	VI	6	M	81	17.5	E3/E3	WB, QPCR	31,26
No. 70	AD	VI	NA	F	63	28	E3/E4	WB, QPCR	812,66
No. 71	AD	VI	5	F	92	60	E3/E4	WB, QPCR	937,69
No. 72	AD	VI	5	M	76	10	E3/E4	WB, QPCR	427,17
No. 73	AD	VI	5	M	73	45	E3/E4	WB, QPCR	572,01
No. 74	AD	VI	4	M	83	34	E3/E4	WB, QPCR	187,54
No. 75	AD	VI	NA	M	70	6	E4/E4	WB, QPCR	625,13
No. 76	AD	VI	NA	F	81	8	E3/E3	WB	
No. 77	AD	VI	4	F	72	24	E3/E3	WB, IHC	
No. 78	AD	VI	4	F	70	45	E3/E4	WB, QPCR, IHC	894,41
No. 79	AD	VI	4	F	60	24	E3/E3	WB, QPCR, IHC	41,68
No. 80	AD	VI	4	F	76	20	E2/E4	WB, QPCR	45,15
No. 81	AD	VI	2	F	91	5.5	E3/E4	WB, QPCR, IHC	69,46
No. 82	AD	VI	5	F	70	21	E3/E3	WB	
No. 83	AD	VI	2	M	84	7	E3/E3	WB, QPCR	34,73
No. 84	AD	VI	5	M	65	30	E3/E3	WB, QPCR, IHC	20,84

No. 85	AD	V	NA	F	88	6	E3/E3	WB	
No. 86	AD	V	NA	M	78	24	E3/E3	WB	
No. 87	AD	V	NA	NA	NA	NA	E3/E3	WB	
No. 88	AD	VI	5	F	89	7	E2/E4	WB, QPCR, IHC	38,20
No. 89	AD	VI	5	F	73	22	E4/E4	WB, IHC	
No. 90	AD	VI	NA	NA	82	4.5	E3/E4	WB	
No. 91	AD	VI	NA	NA	76	10	E3/E3	WB	
No. 92	AD	V	NA	M	74	4	E4/E4	WB	
No. 93	FAD *APP* G2149A	VI	5	F	56	NA	E3/E3	WB, QPCR	83,35
No. 94	FAD *PSEN1* R35E, E120D	VI	5	F	49	14.5	E3/E4	WB, QPCR	166,70
No. 95	DSAD	VI	5	F	57	ND	E3/E3	IHC	

Table S2. Human cases analysed in this study.

The neuropathological staging of AD patients was determined according to Braak staging for NFT scores [1] and to Thal staging for amyloid plaque scores [4]. AD: Alzheimer Disease. FAD: familial Alzheimer disease. DSAD: Down syndrome with Alzheimer disease. PMD: *post-mortem* delay. NA: not available. Frozen tissues of T1 isocortex were analyzed by quantitative polymerase chain reactions (qPCR) or by western blotting (WB). Paraffin embedded tissues of T1 isocortex and hippocampus were analysed by immunohistochemistry (IHC). *APOE* genotyping was determined only for the cases with informed consent for genomic analyses. *SYNJ1* mRNA expression normalized to the average of pPib, RNF4 and PolR2A is shown. 100 % was given to the average of normalized SYNJ1 mRNA in the control group.

References

1 Braak H, Braak E (1991) Neuropathological stageing of Alzheimer-related changes. Acta Neuropathol 82: 239-259

2 Cossec JC, Lavaur J, Berman DE, Rivals I, Hoischen A, Stora S et al. (2012) Trisomy for synaptojanin1 in Down syndrome is functionally linked to the enlargement of early endosomes. Hum Mol Genet 21: 3156-3172

3 Herrera F, Chen Q, Fischer WH, Maher P, Schubert DR (2009) Synaptojanin-1 plays a key role in astrogliogenesis: possible relevance for Down's syndrome. Cell Death Differ 16: 910-920

4 Thal DR, Rub U, Orantes M, Braak H (2002) Phases of A beta-deposition in the human brain and its relevance for the development of AD. Neurology 58: 1791-1800
